# *Plasmodium vivax* and SARS-CoV-2 co-infection in Venezuelan pregnant women: a case series

**DOI:** 10.1186/s12936-023-04442-4

**Published:** 2023-01-07

**Authors:** Fhabián S. Carrión-Nessi, Daniela L. Mendoza-Millán, Óscar D. Omaña-Ávila, Sinibaldo R. Romero, Augusto Moncada-Ortega, Mary Lopez-Perez, Jaime R. Torres, Óscar Noya-González, David A. Forero-Peña

**Affiliations:** 1 Biomedical Research and Therapeutic Vaccines Institute, Ciudad Bolivar, Venezuela; 2 “Dr. Francisco Battistini Casalta” Health Sciences School, University of Oriente – Bolivar Nucleus, Ciudad Bolivar, Venezuela; 3 “Luis Razetti” School of Medicine, Central University of Venezuela, Caracas, Venezuela; 4 Medical Scientist Training Program (MD/PhD), University of Minnesota Medical School, Minneapolis, USA; 5 “José María Vargas” School of Medicine, Central University of Venezuela, Caracas, Venezuela; 6 Centre for Medical Parasitology, Department of Immunology and Microbiology, Faculty of Health and Medical Sciences, University of Copenhagen, Copenhagen, Denmark; 7 Infectious Diseases Section, “Dr. Félix Pifano” Tropical Medicine Institute, Central University of Venezuela, Caracas, Venezuela; 8 Centro Para Estudios Sobre Malaria, “Dr. Arnoldo Gabaldón” High Studies Institute, Caracas, Venezuela; 9 Infectious Diseases Department, University Hospital of Caracas, Caracas, Venezuela

**Keywords:** Case series, COVID-19, Malaria, *Plasmodium vivax*, SARS-CoV-2, Venezuela

## Abstract

**Background:**

Malaria‐endemic areas are not spared from the impact of coronavirus disease 2019 (COVID-19), leading to co-infection scenarios where overlapping symptoms impose serious diagnostic challenges. Current knowledge on *Plasmodium* spp. and severe acute respiratory syndrome coronavirus 2 (SARS-CoV-2) co‐infection in pregnant women remains limited, especially in Latin America, where *Plasmodium vivax* infection is highly prevalent.

**Methods:**

This is a case series of five pregnant women with *P. vivax* and SARS-CoV-2 co-infection hospitalized in two main malaria referral centers of the Capital District and Bolivar state, Venezuela between March 13, 2020 and December 31, 2021.

**Results:**

Clinical and laboratory data from five pregnant women with a mean age of 22 years were analyzed; three of them were in the third trimester of pregnancy. Comorbidities included obesity in two cases, hypertension in one, and asthma in one. Three out of five patients had severe to critical COVID-19 disease. Dry cough, fever, chills, and headache were the most frequent symptoms reported. Laboratory analyses showed elevated aspartate/alanine aminotransferase and creatinine levels, thrombocytopenia, and severe anemia as the most relevant abnormalities. The mean period between symptom onset and a positive molecular test for SARS-CoV-2 infection or positive microscopy for *Plasmodium* spp. was 4.8 ± 2.5 days and 2.8 ± 1.6 days, respectively. The mean hospital stay was 5.4 ± 7 days. Three women recovered and were discharged from the hospital. Two women died, one from cerebral malaria and one from respiratory failure. Three adverse fetal outcomes were registered, two miscarriages and one stillbirth.

**Conclusion:**

This study documented a predominance of severe/critical COVID-19 disease and a high proportion of adverse maternal–fetal outcomes among pregnant women with malaria and COVID-19 co-infection. More comprehensive prospective cohort studies are warranted to explore the risk factors, management challenges, and clinical outcomes of pregnant women with this co-infection.

## Background

The impact of coronavirus disease 2019 (COVID-19) in the world has been unprecedented, particularly affecting low- and middle-income countries where healthcare systems are already weakened and overburden by other diseases such as malaria and arboviral diseases [1, 2]. Pregnant women who live in malaria-endemic areas and get infected by severe acute respiratory syndrome coronavirus 2 (SARS-CoV-2) may be at increased risk of severe COVID-19 or unfavorable disease outcomes if their malaria status is overlooked [3].

Most malaria and COVID-19 co-infections had been reported in Africa and Asia, where patients with *Plasmodium falciparum* and SARS-CoV-2 co-infection were typically oligosymptomatic at presentation with mild to moderate parasitemia, thrombocytopenia, lymphopenia, and elevated bilirubin levels [3, 4]. However, a recent study has shown that co-infection may be associated with higher in-hospital mortality compared to SARS-CoV-2 mono-infection [5]. Current knowledge on malaria and COVID-19 co-infection in pregnant women remains limited [6, 7], especially in Latin America, where *Plasmodium vivax* infection is highly prevalent.

Venezuela remains a malaria breeding ground in the region, representing 34% of cases and 47% of disease-related deaths in America in 2021. Of note, *P. vivax* accounted for 82% of reported cases, followed by *P. falciparum* (13%), and mixed malaria (*P. vivax*/*P. falciparum*; 4%) [8]. This is a case series of pregnant women with *P. vivax* and SARS-CoV-2 co-infection in Venezuela.

## Methods

A retrospective medical record review of all malaria and COVID-19 co-infections in pregnant women registered at the *Centro para Estudios sobre Malaria*, Capital District and “Ruiz y Páez” University Hospital Complex, Bolivar state, Venezuela (March 13, 2020 to December 31, 2021) was conducted. Clinical and laboratory data were obtained from the patients’ medical records. Laboratory tests were carried on the hospital as part of the routine management. Malaria diagnosis by microscopy was performed using Giemsa-stained blood smears, but data on parasite density were not available. SARS-CoV-2 infection was confirmed by reverse transcription polymerase chain reaction (RT-PCR) [9] at the “Rafael Rangel” National Institute of Hygiene (Venezuela).

The severity of COVID-19 was characterized as mild (defined as any of the various signs and symptoms of COVID-19 but no shortness of breath, dyspnea, or abnormal chest imaging), moderate (defined as evidence of lower respiratory disease during clinical assessment or imaging and an oxygen saturation ≥ 94% on room air at sea level), severe (defined as oxygen saturation < 94% on room air at sea level, a ratio of arterial partial pressure of oxygen to fraction of inspired oxygen < 300 mmHg, a respiratory rate > 30 breaths/min, or lung infiltrates > 50%), or critical (defined as respiratory failure, septic shock, and/or multiple organ dysfunction), according to the National Institutes of Health (United States) guidelines [10].

## Results

A total of 253 and 2547 medical records of malaria cases at the *Centro para Estudios sobre Malaria* and “Ruiz y Páez” University Hospital Complex, respectively, were reviewed. The demographic, clinical, and paraclinical profiles of five pregnant women with confirmed co-infection by *P. vivax* and SARS-CoV-2 were recorded (Tables 1, 2). All pregnant woman were managed as inpatients regardless of their clinical condition, two of them had critical COVID-19 disease, while one of each had mild, moderate, or severe COVID-19 disease. None of them received remdesivir or tocilizumab for the management of COVID-19. Per protocol, all patients with severe/critical COVID-19 disease received steroids, supplemental oxygen, and thrombosis prophylaxis. All pregnant women also received anti-malarial treatment according to the latest national protocol [11]. The mean period between symptom onset and a positive test was 4.8 ± 2.5 days for SARS-CoV-2 infection and 2.8 ± 1.6 for *Plasmodium* spp. The mean time lag between the two diagnoses was 3.8 ± 1.3 days. Although malaria confirmation preceded the diagnosis of COVID-19 by three to five days, none of COVID-19 cases were considered hospital-acquired.

**Table 1:** Demographic and clinical profiles of pregnant women co-infected with *P. vivax* and SARS-CoV-2

Variables	Patient 1	Patient 2	Patient 3	Patient 4	Patient 5
*Demographical*					
Age, years	25	28	21	15	20
Occupation	Cook in mining camp	Housewife	Housewife	Student	Housewife
Trimester of pregnancy	Third trimester	Third trimester	Second trimester	Second trimester	Third trimester
Previous pregnancies, no	2	2	2	0	1
*Clinical*					
Comorbidities	Obesity	Hypertension	Obesity	No	Asthma
COVID-19 severity	Moderate	Severe	Critical	Critical	Mild
Previous malaria episodes, no	7	3	1	0	2
Past malaria parasite	*P. vivax*	*P. vivax*	*P. vivax*	–	Mixed
Maternal complications	No	Preeclampsia	Cerebral malaria	Hyperemesis gravidarum	No
Fetal complications	Stillbirth	No	Miscarriage	Miscarriage	No
Maternal outcome	Discharge	Discharge	Death	Death	Discharge
Fetal outcome (gestational age)	Death (week 29)	Live birth (week 30)	Death (week 20)	Death (week 16)	Live birth (week 38)
Newborn’s weight, g	–	1305	–	–	2949
Newborn’s APGAR at 5^th^ minute, points	–	4	–	–	9
Hospital stay, days	1	17	2	0	7

*COVID-19* coronavirus disease 2019

**Table 2:** Paraclinical profile of pregnant women co-infected with *P. vivax* and SARS-CoV-2

Variables	Patient 1	Patient 2	Patient 3	Patient 4	Patient 5
*Paraclinicals*					
Hemoglobin, g/dL	10.8	9.3	6.2	9.7	11.2
White blood cells, × 10^3^/mL	5.6	11.9	13	9	9.6
Neutrophils, × 10^3^/mL	3.42	9.42	10.4	6.75	N/A
Lymphocytes, × 10^3^/mL	2.18	2.50	2.60	2.25	N/A
NLR	1.6	3.8	4.0	3.0	N/A
Platelets, × 10^3^/mL	87	177	121	330	146
Glycemia, mg/dL	N/A	44	N/A	102	73
Urea, mg/dL	N/A	38	N/A	82	37
Creatinine, mg/dL	N/A	2.14	N/A	1.59	0.6
AST, U/L	65	48	N/A	60	23
ALT, U/L	50	24	N/A	68	13

*NLR* neutrophil-to-lymphocyte ratio, *AST* alanine aminotransferase, *AST* aspartate aminotransferase, *N/A* not available

Patients’ age ranged from 15 to 28 years (mean 22 ± 5 years). Three of them were in the third trimester of pregnancy, two suffered from obesity, one was hypertense, and one was asthmatic. Dry cough (4/5), fever (3/5), chills (3/5), and headache (2/5) were the most frequent symptoms reported on admission. Less common symptoms included dyspnea (1/5), arthralgia (1/5), and vomiting (1/5). Mild elevations of hepatic enzymes, creatinine, and urea serum levels were observed. Likewise, patients exhibited mild alterations in the number of platelets, leucocytes, and neutrophils. Only one had severe anemia and none of them had lymphopenia. Three pregnant women recovered and were discharged from the hospital. Two women died, one from cerebral malaria and one from respiratory failure. Three adverse fetal outcomes were registered, two miscarriages and one stillbirth. The mean hospital stay was 5.4 ± 7 days.

## Discussion

This analysis describes a case series of five pregnant women with malaria and COVID-19 co-infection in Venezuela, with a predominance of severe/critical COVID-19, including two maternal–fetal deaths and one stillbirth. Although some reports showed that *P. vivax* and SARS-CoV-2 co-infection had a mainly mild clinical course [12–14], even in pregnant women [7, 15], a high proportion of moderate/severe COVID-19 disease among co-infected patients in Venezuela was recently reported [16].

Malaria and COVID-19 symptoms may overlap and delay the diagnosis of co-infection. Symptoms on admission in this case series were similar to those previously reported [3, 15], being anemia and thrombocytopenia two common laboratory alterations among pregnant women with this co-infection [3]. It is unclear whether laboratory parameters could help discriminate malaria and COVID-19 co-infection [3], since some laboratory alterations may be more common in SARS-CoV-2 mono-infected than in co-infected patients and yet other more frequent in malaria mono-infected patients [17–20].

Since malaria and COVID-19 have different incubation periods, one infection could potentiate the subsequent one. To the authors’ knowledge, this has not been addressed or reviewed at full length in other studies. Indeed, only few studies have examined this potential relationship [21]. A unifying theme of the pathogenesis of both diseases is oxidative stress via 8-iso-prostaglandin F2α (8-iso-PGF2α). Muhammad et al. [22], for example, reported in 2020 that 8-iso-PGF2α levels were significantly higher in patients co-infected with malaria and COVID-19 as those only infected with COVID-19; further, the levels of 8-iso-PGF2α were proportional to malaria parasite density in the infected patients. Therefore, albeit more studies are warranted, a tentative potentiation effect could be inferred in this rare co-infection setting. Osei et al. [21] have postulated that T-cell exhaustion or inadequate B-cell response could be the culprit.

A higher risk of adverse outcomes in pregnant women with either *Plasmodium* spp. [23] or SARS-CoV-2 [24, 25] have been reported, but not so much for co-infected pregnant women, including co-infection with *P. vivax* [7, 15] and *Plasmodium ovale* [6]. No similar reports for *P. falciparum* co-infection were found. Pregnancy-associated malaria increases the risk of anemia, stillbirth, low birth weight, and maternal–fetal death due to systemic and placental inflammatory responses and microvascular dysfunction [23, 26]. SARS-CoV-2 mono-infection during pregnancy is also associated with an increased risk of pre-eclampsia, preterm birth, low birth weight, stillbirth, and mechanical ventilation [27, 28]. Severe placentitis and vascular dysfunction have been described, leading to long-term multisystemic defects in exposed infants [24]. Thus, delayed care may have life-threatening consequences [15], as was evident in three of the five patients of this case series.

Recently, it was documented a high prevalence of maternal–fetal complications in Venezuela caused mainly by *P. vivax* [29], traditionally described as benign. On the other hand, while the clinical behavior of patients with COVID-19 during the first wave in Venezuela showed a high overall and in-hospital mortality [30], the clinical outcome of the first locally recorded pregnant woman infected with SARS-CoV-2 and her child was favorable [31]. However, two recent studies reported a high prevalence of maternal–fetal events among Venezuelan pregnant women with COVID-19 [32, 33]. Therefore, simultaneous infection with these two pathogens could create a favorable setting leading to increased maternal–fetal risk in pregnant women.

Due to the retrospective nature of this work, several limitations were unavoidable. Clinical and paraclinical information was incomplete for some patients and we did not collect specific outcomes of COVID-19-associated post-acute complications, such as thrombotic events. Moreover, there was an inherent selection bias, as all analyzed cases came from the primary malaria-endemic region of the country and included only hospitalized cases, so these severe maternal–fetal results cannot be extrapolated to populations with different epidemiological and clinical characteristics. As a strength, all malaria and COVID-19 co-infections in the current case series were confirmed by microscopy and RT-PCR, respectively. In the case of the dead pregnant woman with cerebral malaria, although no co-infection by *P. falciparum* was detected in the Giemsa-stained peripheral blood smears, such possibility cannot be ruled out, since blood samples not taken during the febrile peak may not reveal low level circulating parasites, as most of them remain adhered to vascular endothelia. Because a high level of false-positive results with the SARS-CoV-2 serological assay has been identified in highly malaria-endemic areas [34, 35], routine use of serological assay may overestimate the level of exposure and immunity of the population to SARS-CoV-2 in malaria-endemic countries [36].

## Conclusions

This study documented a predominance of severe/critical COVID-19 diseases, as well as a high proportion of adverse maternal–fetal outcomes among malaria co-infected pregnant women. COVID-19 has become an unprecedented challenge to healthcare systems worldwide and has affected malaria control programs by delaying the distribution of insecticide-treated nets, and disrupting early diagnosis and drug supplies, among others [37, 38]. Malaria-endemic areas in Venezuela are not spared from those challenges [39] and delays in malaria diagnosis during pregnancy could favor mortality and severe complications [40]. Thus, the need for timely diagnosis to ensure an appropriate clinical management is emphasized. Furthermore, large prospective cohort studies are warranted to explore the risk factors, clinical outcomes, management challenges, and outcome of pregnant women with this co-infection.

## Data Availability

All data and materials in this article are included in the manuscript.

## Abbreviations

COVID-19: Coronavirus disease 2019
SARS-CoV-2: Severe acute respiratory syndrome coronavirus 2
RT-PCR: Reverse transcription polymerase chain reaction
8-iso-PGF2α: 8-iso-prostaglandin F2α
